# A Population-Based Case–Control Study of Extreme Summer Temperature and Birth Defects

**DOI:** 10.1289/ehp.1104671

**Published:** 2012-06-27

**Authors:** Alissa R. Van Zutphen, Shao Lin, Barbara A. Fletcher, Syni-An Hwang

**Affiliations:** 1 Bureau of Environmental and Occupational Epidemiology, New York State Department of Health, Albany, New York, USA; 2 Department of Epidemiology and Biostatistics, University at Albany, Rensselaer, New York, USA

**Keywords:** birth defects, climate change, congenital cataracts, heat, temperature

## Abstract

Background: Although hyperthermia is a recognized animal teratogen and maternal fever has been associated with birth defects in humans, data on the relationship between high environmental temperatures and birth defects are limited.

Objective: To determine whether pregnancies are potentially vulnerable to the weather extremes anticipated with climate change, we evaluated the relationship between extreme summer temperature and the occurrence of birth defects.

Methods: We performed a population-based case–control study by linking the New York State Congenital Malformations Registry to birth certificates for the years 1992–2006. We selected nonmalformed infants from a 10% random sample of live births as controls. We assigned meteorologic data based on maternal residence at birth, summarized universal apparent temperature (UAT; degrees Fahrenheit) across the critical period of embryogenesis, and estimated adjusted odds ratios (aOR) and 95% confidence intervals (CI) with multivariable logistic regression, controlling for confounders available on the birth certificate.

Results: Among 6,422 cases and 59,328 controls that shared at least 1 week of the critical period in summer, a 5-degree increase in mean daily minimum UAT was significantly associated with congenital cataracts (aOR = 1.51; 95% CI: 1.14, 1.99). Congenital cataracts were significantly associated with all ambient temperature indicators as well: heat wave, number of heat waves, and number of days above the 90th percentile. Inconsistent associations with a subset of temperature indicators were observed for renal agenesis/hypoplasia (positive) and anophthalmia/microphthalmia and gastroschisis (negative).

Conclusions: We found positive and consistent associations between multiple heat indicators during the relevant developmental window and congenital cataracts which should be confirmed with other data sources.

Hyperthermia is a well-known animal teratogen, and maternal fever has been associated with birth defects in human studies (Edwards 2006; Edwards et al. 1995; Graham 2005; Graham et al. 1998; Warkany 1986). In all species, the teratogenic effect of the hyperthermic insult depends on timing, intensity, and duration of exposure, and the central nervous system appears to be most vulnerable. Most anomalies detected in animal studies have been observed in clinical and epidemiologic studies of maternal fever and febrile illness, including neural-tube defects, microphthalmia, congenital cataracts, abdominal wall defects, congenital heart defects, microcephaly, limb defects, craniofacial malformations, and renal defects (Edwards 2006). In a meta-analysis of epidemiologic studies of maternal first trimester hyperthermia and neural-tube defects, a statistically significant overall odds ratio (OR) was reported (Moretti et al. 2005). Furthermore, associations with maternal fever were attenuated after adjustment for the use of antipyretics, suggesting that the fever was teratogenic and not the underlying illness (Czeizel et al. 2007; Hashmi et al. 2010; Oster et al. 2011; Vogt et al. 2005).

There is a paucity of data on the teratogenicity of external heat-generating sources or hot environments, which may increase maternal core temperature during pregnancy, and the exposure assessments for these studies are typically based on postpartum maternal interviews about environmental conditions during early pregnancy. Existing data support a possible association of neural-tube defects with hot tub and sauna use during early pregnancy (Chambers 2006; Suarez et al. 2004), but not with hot environments (Suarez et al. 2004). In studies of congenital heart defects, no associations were observed for hot tub, bath, or sauna use or exposures to hot environments in early pregnancy (Judge et al. 2004; Tikkanen and Heinonen 1991). The few studies of electric blanket, heated waterbed, or heated mattress pad use have not shown strong evidence of associations with neural-tube defects or oral clefts (Dlugosz et al. 1992; Milunsky et al. 1992; Shaw et al. 1999).

It is likely that temperatures will increase and extreme weather events will become more common with climate change (Intergovernmental Panel on Climate Change 2007). Because Northeasterners are less accustomed to heat, they are more likely to be adversely affected by extreme heat events (Luber and McGeehin 2008). Although it is biologically plausible that extreme ambient outdoor temperatures during pregnancy could raise maternal core temperatures and cause birth defects, few studies have assessed the relationship between high environmental temperatures and birth defects. Our objective was to evaluate the relationship between extreme heat and the risk of birth defects using the population-based New York State Congenital Malformations Registry (CMR; Albany, NY) to determine whether pregnant women and their infants in New York State are potentially vulnerable to effects of climate change.

## Methods

*Study population and data sources.* The source population was all live births to residents of upstate New York (New York State, excluding New York City) from 1992 through 2006. Appropriate institutional review board approvals were granted to access New York State birth certificate data from Vital Records (New York State Department of Health, Albany, NY) and birth defects data from the CMR. The birth certificate contains data on maternal and infant characteristics, such as maternal age, race/ethnicity, education, date of last menses, prenatal care, and maternal behavioral characteristics (e.g., smoking), infant date of birth, sex, birth weight, and gestational age. Roohan et al. (2003) assessed the validity of information reported on the New York State birth certificate using medical records and reported high specificity (91–100%) for most data elements and high sensitivity for information such as birth weight (100%) and maternal lifestyle (86–100%). The CMR is a population-based registry that receives mandated reports on children who were born in New York State and were diagnosed with birth defects, metabolic defects, or chromosomal anomalies up until 2 years of age from hospitals and physicians. Onsite hospital medical record audits indicated that CMR reports were > 90% correct (Wang et al. 2010), an ascertainment level comparable to that of the Metropolitan Atlanta (GA) Congenital Defects Program, an active surveillance system that is regarded as the “gold standard” (Honein and Paulozzi 1999).

*Study design and data linkage.* We used a case–control study design to examine the association between high ambient temperature during the critical period of embryogenesis and the prevalence of birth defects. Cases were upstate New York live births with birth defects for the 1992–2006 study period, and controls were a 10% random sample of upstate New York live births without birth defects for the same period. Individual birth defects records were linked with birth certificate records.

*Outcome assessment.* Using *International Classification of Diseases, 9th Revision, Clinical Modification* (ICD-9-CM; Centers for Disease Control and Prevention 2011a) diagnoses codes from the CMR records, birth defect cases were classified into the 45 birth defects categories that meet the reporting standards of the National Birth Defects Prevention Network (NBDPN 2010). Of these, we selected the 28 groups of major birth defects within the six body systems with prior animal or human studies suggesting an association with heat: central nervous system (e.g., neural-tube defects, microcephaly), eye (e.g., microphthalmia, congenital cataracts), cardiovascular, craniofacial, renal, and musculoskeletal defects (e.g., abdominal wall defects, limb defects).

**Figure 1:**
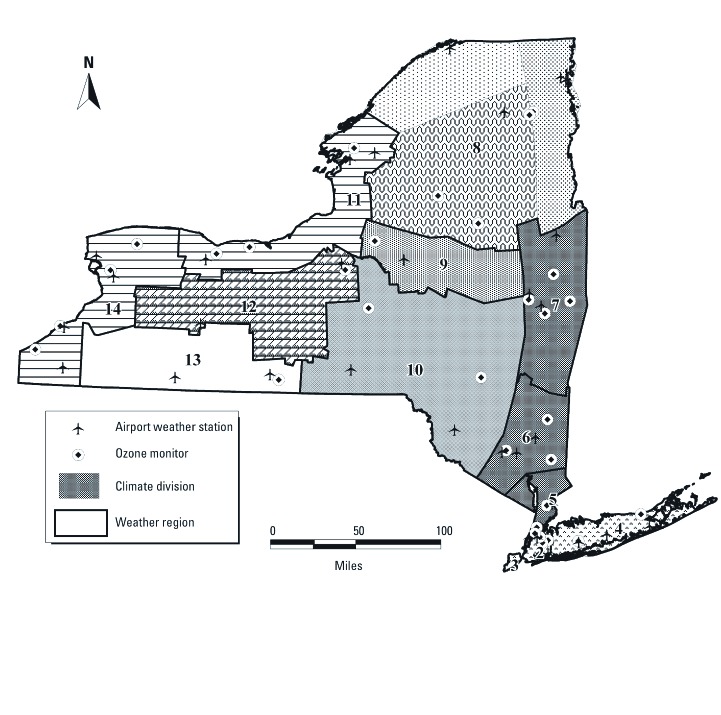
Weather regions, climate divisions, airport weather stations, and ozone monitors in New York State.

*Exposure assessment.* We divided New York State into 14 weather regions by overlaying and merging 10 New York State climate divisions defined by the National Climatic Data Center (2009) with the 11 ozone regions developed for New York State by Chinery and Walker (2009) to create 14 regions of relatively homogeneous weather and ozone exposures (Figure 1). Each weather region was assigned a daily average value of temperature (minimum, mean, and maximum in degrees Fahrenheit), barometric pressure, dew point, and wind speed based on hourly meteorologic observations obtained from the National Center for Atmospheric Research (2009) for 18 first-order airport weather stations maintained by the National Weather Service or the Federal Aviation Administration. Universal apparent temperature (UAT) was calculated based on Steadman’s formula, using temperature, vapor pressure, and wind speed (Steadman 1984). About 94% of residential addresses were geocoded to ZIP code using Map Marker Plus and MapInfo Professional (Pitney Bowes Business Insight, Troy, NY) and assigned to one of the 11 upstate New York weather regions. Less than 6% of records were excluded from the analysis due to inadequate address information.

We estimated date of conception by adding 14 days to the date of last menses, and we estimated day of pregnancy based on this reference point. Daily average values of meteorologic variables were merged with the birth record based on estimated day of pregnancy and weather region. To examine the effects of extreme heat in summer, we restricted the analyses to summer months of June, July, and August. We focused on weeks 3–8 postconception, during organogenesis, as the critical period (Sadler 2004). Because UAT is a better indicator of thermal stress on the human body (Steadman 1984), we averaged the daily minimum, mean, and maximum UAT across the critical period for each case and control birth. We created exposure indices to capture frequency and duration of extreme heat events using the mean UAT distributions within each weather region to account for potential differential acclimatization by region. We defined a heat wave as at least 3 consecutive days with mean UAT above the 90th percentile. We counted the number of heat waves and the number of days above the 90th percentile. To look at finer exposure windows, we also averaged the daily minimum, mean, and maximum UAT for each week of the first trimester for each birth.

*Confounders and effect modifiers.* Maternal age (< 20, 20–34, ≥ 35 years), education level (< 12, 12–15, ≥ 16 years), race (white, black, other), ethnicity (Hispanic, non-Hispanic), total previous live births (0, 1, ≥ 2), prenatal care (modified Kessner Index: adequate, intermediate, inadequate), smoking (yes, no), alcohol consumption (yes, no), multiple birth (yes, no), and infant sex (male, female) were derived from the birth certificates and considered as potential confounders and effect modifiers. Additionally, weather region was categorized based on maternal residential address.

*Statistical analysis.* For the subset of births with at least 1 week of the critical period of embryogenesis within summer, we used unconditional logistic regression to estimate the association between each heat exposure indicator and selected birth defects group. Crude prevalence ORs and 95% confidence intervals (CIs) were calculated, and confounding and interaction were assessed using stratified analyses. Multivariable unconditional logistic regression was used to calculate adjusted ORs (aORs) and 95% CIs. Potential confounders were dropped out of the model in a reverse fashion, and a change in the effect estimate of > 10% was used as the criterion for keeping the variable in the model. Final models were adjusted for year, weather region, maternal age, ethnicity, education level, adequacy of prenatal care, and smoking status. For the associations between heat waves and each birth defects group, we performed stratified analyses by weather region, maternal race, ethnicity, age, education, adequacy of prenatal care, and smoking status. In our analyses, we used a *p*-value of 0.05.

To control for potential detection bias due to increased surveillance among preterm infants, we performed subanalyses among term infants for congenital cataracts (Prakalapakorn et al. 2010) and certain heart defects (i.e., patent ductus arteriousus, atrial septal defect, ventricular septal defect) (Tanner et al. 2005).

## Results

A total of 6,422 selected birth defects cases and 59,328 controls that shared at least 1 week of the critical period of embryogenesis in summer were included in the analyses of upstate New York resident live births in the 1992–2006 study period. Distributions of maternal age, education, race, total previous live births, adequacy of prenatal care, smoking, alcohol use, multiple birth, low birth weight, preterm birth, region, and year of birth were significantly different between cases and controls (Table 1). Distributions of daily universal apparent temperature in summer by weather region are displayed in Table 2.

**Table 1:** Characteristics of live births with at least 1 week of the critical period of embryogenesis during June–August, upstate New York, 1992–2006 [n (%)].

Characteristic	Cases (n = 6,422)	Controls (n = 59,328)
Maternal age (years)*
< 20	574 (8.9)	4,607 (7.8)
20–34	4,765 (74.2)	44,769 (75.5)
≥ 35	1,081 (16.8)	9,930 (16.7)
Maternal education (years)*
< 12	1,049 (16.7)	8,424 (14.5)
12–15	3,569 (56.7)	32,044 (55.0)
≥ 16	1,672 (26.6)	17,834 (30.6)
Maternal race*
White	5,323 (83.3)	50,198 (85.0)
Black	785 (12.3)	6,121 (10.4)
Other race	286 (4.5)	2,755 (4.7)
Maternal ethnicity
Hispanic	690 (9.3)	5,565 (9.4)
Non-Hispanic	5,822 (90.7)	53,763 (90.6)
Total previous live births*
0	2,675 (41.7)	23,925 (40.3)
1	2,063 (32.1)	20,017 (33.7)
≥ 2	1,682 (26.2)	15,382 (25.9)
Modified Kessner Prenatal Care Index*
Adequate care	4,160 (64.8)	39,764 (67.0)
Intermediate care	1,458 (22.7)	13,931 (23.5)
Inadequate care	804 (12.5)	5,633 (9.5)
Maternal smoking^a^
Yes	1,155 (18.2)	9,145 (15.7)
No	5,197 (81.8)	49,303 (84.4)
Maternal alcohol use*
Yes	82 (1.3)	544 (0.9)
No	6,263 (98.7)	57,822 (99.1)
Multiple birth*
Yes	382 (6.0)	1,969 (3.3)
No	6,039 (94.1)	57,359 (96.7)
Infant sex
Male	3,262 (50.8)	29,866 (50.3)
Female	3,160 (49.2)	29,462 (49.7)
Low birth weight (g)*
< 2,500	1,275 (19.9)	3,740 (6.3)
≥ 2,500	5,140 (80.1)	55,541 (93.7)
Preterm birth (weeks)*
< 37	1,495 (23.3)	5,769 (9.7)
≥ 37	4,927 (76.7)	53,559 (90.3)
Weather region*
Long Island	1,699 (26.5)	15,064 (25.4)
Westchester/Rockland	601 (9.4)	6,730 (11.3)
Hudson Valley–South	365 (5.7)	4,144 (7.0)
Hudson Valley–North	496 (7.7)	4,771 (8.0)
Adirondack and North	154 (2.4)	1,825 (3.1)
Mohawk Valley	233 (3.6)	2,116 (3.6)
Binghamton	480 (7.5)	4,179 (7.0)
Great Lakes–Rochester	589 (9.2)	6,279 (10.6)
Central Lakes	549 (8.6)	4,967 (8.4)
Western Plateau	230 (3.6)	1,774 (3.0)
Great Lakes-Buffalo	1,026 (16.0)	7,479 (12.6)
Month of conception
April	972 (15.1)	9,347 (15.8)
May	1,615 (25.2)	14,662 (24.7)
June	1,639 (25.5)	14,947 (25.2)
July	1,683 (26.2)	15,582 (26.3)
August	513 (8.0)	4,790 (8.1)
Year of birth*
1992	446 (6.9)	4,519 (7.6)
1993	491 (7.7)	4,465 (7.5)
1994	406 (6.3)	4,314 (7.3)
1995	408 (6.4)	4,104 (6.9)
1996	432 (6.7)	3,977 (6.7)
1997	382 (6.0)	4,002 (6.8)
1998	413 (6.4)	3,953 (6.7)
1999	399 (6.2)	3,933 (6.6)
2000	397 (6.2)	3,925 (6.6)
2001	384 (6.0)	3,865 (6.5)
2002	400 (6.2)	3,805 (6.4)
2003	424 (6.6)	3,746 (6.3)
2004	468 (7.3)	3,577 (6.0)
2005	456 (7.1)	3,548 (6.0)
2006	516 (8.0)	3,595 (6.1)
*Significant difference between cases and controls based on chi-square test (p ≤ 0.05).		

**Table 2:** Regional distributions of daily minimum, mean, and maximum UAT in summer, upstate New York, 1991–2006.

Weather region	Daily minimum UAT					Daily mean UAT					Daily maximum UAT				
	Mean ± SD	Minimum	Maximum	Mean ± SD	Minimum	Maximum	Mean ± SD	Minimum	Maximum
4. Long Island	66.1 ± 8.6	40.8	91.4	75.0 ± 8.3	52.9	99.9	83.4 ± 8.9	57.0	111.1
5. Westchester/Rockland	63.9 ± 7.7	41.2	90.5	73.6 ± 8.1	53.1	99.4	83.1 ± 9.2	55.0	111.3
6. Hudson Valley–South	61.5 ± 7.9	34.8	88.4	73.0 ± 7.9	52.2	97.9	84.0 ± 9.3	53.5	109.4
7. Hudson Valley–North	59.2 ± 7.6	36.2	84.4	70.6 ± 7.4	49.7	94.8	81.5 ± 8.9	53.5	106.6
8. Adirondack and North	57.9 ± 8.0	29.8	86.2	69.2 ± 7.8	45.0	94.5	80.1 ± 9.2	52.0	106.6
9. Mohawk Valley	59.1 ± 7.9	12.0	86.9	69.6 ± 7.7	46.4	95.4	80.1 ± 9.2	52.0	108.0
10. Binghamton	58.5 ± 7.2	31.5	81.2	67.7 ± 7.3	43.5	90.4	77.0 ± 8.6	51.0	102.9
11. Great Lakes–Rochester	58.3 ± 8.1	33.8	85.2	69.3 ± 7.8	45.2	95.1	79.5 ± 8.7	55.3	104.3
12. Central Lakes	60.5 ± 7.8	31.6	87.9	71.3 ± 8.0	47.2	95.7	81.5 ± 9.3	53.0	108.1
13. Western Plateau	56.6 ± 7.8	30.0	83.2	69.2 ± 7.4	45.5	93.8	81.6 ± 9.0	54.0	109.1
14. Great Lakes–Buffalo	61.1 ± 8.0	36.2	88.4	71.1 ± 7.9	43.0	93.4	80.5 ± 8.8	50.0	105.5
Overall: upstate New York	60.2 ± 8.3	12.0	91.4	70.9 ± 8.1	43.0	99.9	81.1 ± 9.2	50.0	111.3

A 5-degree increase in the mean daily minimum UAT in the critical period was significantly associated with an increased occurrence of congenital cataracts (aOR = 1.51; 95% CI: 1.14, 1.99) and renal agenesis/hypoplasia (aOR = 1.17; 95% CI: 1.00, 1.37) and a reduced occurrence of anophthalmia/microphthalmia (aOR = 0.71; 95% CI: 0.54, 0.94) (Table 3). Associations with mean and maximum UAT were similar for these outcomes, though not statistically significant for renal agenesis/hypoplasia. No statistically significant associations were estimated for UAT and central nervous system, cardiovascular, craniofacial, or musculoskeletal birth defect groups.

**Table 3:** Associations between selected birth defects and mean daily universal apparent temperature during the critical period of embryogenesis, upstate New York, 1992–2006.

Birth outcome group	*n*	Daily minimum UAT				Daily mean UAT				Daily maximum UAT			
		Mean ± SD	5°F increase		Mean ± SD	5°F increase		Mean ± SD	5°F increase	
			OR^a^ (95% CI)			OR^a^ (95% CI)			OR^a^ (95% CI)	
Controls	59,328	59.1 ± 6.2	—		69.4 ± 5.9	—		79.2 ± 5.7	—	
Central nervous system
Anencephalus	21	56.9 ± 3.6	0.94	(0.63, 1.42)	67.4 ± 3.8	0.93	(0.61, 1.43)	77.6 ± 4.0	0.95	(0.62, 1.46)
Spina bifida without anencephalus	114	58.6 ± 5.8	1.12	(0.92, 1.35)	68.9 ± 5.4	1.10	(0.90, 1.34)	78.8 ± 5.2	1.08	(0.89, 1.32)
Hydrocephalus without spina bifida	311	59.3 ± 6.2	1.06	(0.95, 1.18)	69.6 ± 5.7	1.07	(0.95, 1.20)	79.4 ± 5.4	1.07	(0.95, 1.19)
Encephalocele	25	58.0 ± 6.1	0.92	(0.64, 1.32)	68.3 ± 5.9	0.92	(0.63, 1.33)	78.2 ± 5.9	0.93	(0.64, 1.35)
Microcephalus	199	58.5 ± 6.9	0.93	(0.82, 1.06)	68.7 ± 6.5	0.93	(0.81, 1.06)	78.4 ± 6.3	0.92	(0.80, 1.05)
Eye
Anophthalmia/microphthalmia	34	58.2 ± 6.2	0.71	(0.54, 0.94)*	68.2 ± 6.2	0.70	(0.53, 0.93)*	77.8 ± 6.4	0.70	(0.52, 0.93)*
Congenital cataract	75	60.5 ± 5.1	1.51	(1.14, 1.99)*	71.0 ± 4.9	1.47	(1.11, 1.94)*	81.0 ± 4.9	1.45	(1.10, 1.90)*
Cardiovascular
Common truncus	9	57.2 ± 8.2	0.99	(0.53, 1.86)	67.7 ± 7.7	1.02	(0.52, 1.98)	77.7 ± 7.2	1.07	(0.54, 2.11)
Transposition of great arteries	68	58.7 ± 5.7	1.04	(0.82, 1.33)	69.4 ± 5.4	1.07	(0.84, 1.37)	79.6 ± 5.3	1.08	(0.84, 1.37)
Tetralogy of Fallot	106	59.3 ± 6.1	0.99	(0.83, 1.19)	69.5 ± 5.8	0.98	(0.82, 1.18)	79.2 ± 5.5	0.98	(0.82, 1.18)
Ventricular septal defect	1,579	59.0 ± 6.2	1.00	(0.96, 1.05)	69.5 ± 5.8	1.01	(0.96, 1.06)	79.2 ± 5.6	1.00	(0.95, 1.05)
Atrial septal defect	822	59.9 ± 6.4	0.96	(0.90, 1.02)	69.8 ± 6.1	0.96	(0.90, 1.02)	79.4 ± 6.0	0.97	(0.90, 1.03)
Endocardial cushion defect	43	58.2 ± 5.3	0.97	(0.73, 1.29)	68.7 ± 5.4	0.97	(0.72, 1.30)	78.7 ± 5.6	0.98	(0.73, 1.32)
Pulmonary valve atresia/stenosis	457	59.3 ± 6.2	1.07	(0.97, 1.17)	69.5 ± 5.8	1.07	(0.98, 1.17)	79.2 ± 5.6	1.07	(0.97, 1.17)
Tricuspid atresia/stenosis	44	58.7 ± 7.1	0.99	(0.75, 1.32)	68.8 ± 6.9	0.97	(0.73, 1.29)	78.4 ± 6.7	0.95	(0.71, 1.27)
Ebstein’s anomaly	23	59.5 ± 4.7	1.06	(0.70, 1.59)	69.9 ± 4.3	1.12	(0.73, 1.71)	79.8 ± 4.3	1.18	(0.76, 1.83)
Aortic valve stenosis	102	59.5 ± 5.8	1.08	(0.89, 1.31)	69.8 ± 5.6	1.08	(0.89, 1.31)	79.6 ± 5.6	1.08	(0.89, 1.31)
Hypoplastic left heart syndrome	96	58.9 ± 6.3	0.99	(0.82, 1.20)	69.3 ± 6.1	0.98	(0.81, 1.19)	79.2 ± 6.0	0.98	(0.81, 1.19)
Patent ductus arteriosus (≥ 2,500 g)	566	59.7 ± 6.1	0.96	(0.89, 1.03)	69.6 ± 5.8	0.95	(0.88, 1.03)	79.0 ± 5.6	0.95	(0.88, 1.03)
Coarctation of aorta	235	59.0 ± 5.9	1.05	(0.92, 1.19)	69.3 ± 5.7	1.06	(0.93, 1.21)	79.2 ± 5.6	1.08	(0.95, 1.24)
Craniofacial
Choanal atresia	99	58.3 ± 6.6	0.90	(0.75, 1.08)	68.7 ± 6.2	0.90	(0.75, 1.08)	78.7 ± 6.0	0.91	(0.76, 1.09)
Cleft palate without cleft lip	340	58.4 ± 6.1	0.99	(0.89, 1.10)	68.8 ± 5.7	0.99	(0.89, 1.10)	78.7 ± 5.6	0.97	(0.87, 1.08)
Cleft lip ± cleft palate	501	58.6 ± 6.4	0.98	(0.90, 1.07)	69.1 ± 6.0	0.98	(0.90, 1.07)	79.1 ± 5.9	0.99	(0.91, 1.07)
Genitourinary
Renal agenesis/hypoplasia	174	59.8 ± 5.8	1.17	(1.00, 1.37)*	70.1 ± 5.5	1.15	(0.99, 1.35)	79.8 ± 5.3	1.13	(0.97, 1.32)
Musculoskeletal
Upper limb reduction	105	58.7 ± 5.6	1.09	(0.89, 1.33)	69.2 ± 5.3	1.11	(0.90, 1.36)	79.3 ± 5.4	1.12	(0.91, 1.38)
Lower limb reduction	85	59.1 ± 6.4	1.10	(0.88, 1.37)	69.4 ± 6.0	1.10	(0.88, 1.38)	79.2 ± 5.7	1.09	(0.87, 1.36)
Gastroschisis	108	58.3 ± 5.6	1.00	(0.83, 1.21)	68.6 ± 5.7	0.98	(0.82, 1.19)	78.5 ± 5.8	0.99	(0.82, 1.19)
Omphalocele	81	59.0 ± 5.7	1.12	(0.89, 1.42)	69.3 ± 5.3	1.12	(0.89, 1.42)	79.1 ± 5.3	1.12	(0.88, 1.41)
aORs were adjusted for maternal age, race, ethnicity, adequacy of prenatal care, smoking, weather region, and year. *Statistically significant (p ≤ 0.05).																	

Congenital cataracts were significantly associated with heat waves (aOR = 1.97; 95% CI: 1.17, 3.32), number of heat waves (aOR = 1.45; 95% CI: 1.04, 2.02), and number of days above the 90th percentile (aOR = 1.09; 95% CI: 1.02, 1.17) (Table 4). The prevalence of gastroschisis was significantly decreased in association with a heat wave event (aOR = 0.48; 95% CI: 0.28, 0.81) and the number of heat waves (aOR = 0.63; 95% CI: 0.43, 0.92). No statistically significant relationships were identified among central nervous system, cardiovascular, craniofacial, or genitourinary birth defect groups.

**Table 4:** Associations between selected birth defects and extreme heat during the critical period of embryogenesis, upstate New York, 1992–2006.

Birth outcome group	*n*	Embryo-days in summer^a^	Heat wave^a^				No. of heat waves				No. of days > 90th percentile			
			*n* (%)	OR^b^ (95% CI)		Mean ± SD	OR^b^ (95% CI)		Mean ± SD	OR^b^ (95% CI)	
Controls	59,328	18,629 (31.4)	—		0.4 ± 0.7	—		3.1 ± 3.8	—	
Central nervous system
Anencephalus	21	2 (9.5)	0.21	(0.04, 1.03)	0.1 ± 0.3	0.25	(0.06, 1.03)	1.7 ± 2.8	0.89	(0.74, 1.05)
Spina bifida without anencephalus	114	38 (33.3)	1.30	(0.82, 2.05)	0.4 ± 0.7	1.12	(0.83, 1.52)	3.2 ± 3.6	1.03	(0.97, 1.09)
Hydrocephalus without spina bifida	311	94 (30.2)	0.95	(0.72, 1.25)	0.4 ± 0.7	0.99	(0.82, 1.20)	3.2 ± 3.8	1.01	(0.97, 1.04)
Encephalocele	25	6 (24.0)	0.57	(0.21, 1.57)	0.4 ± 0.9	1.00	(0.53, 1.86)	3.0 ± 4.8	0.97	(0.86, 1.10)
Microcephalus	199	60 (30.2)	1.10	(0.77, 1.58)	0.4 ± 0.6	0.99	(0.77, 1.28)	3.0 ± 4.0	0.99	(0.95, 1.04)
Eye
Anophthalmia/microphthalmia	34	10 (29.4)	0.65	(0.29, 1.44)	0.4 ± 0.7	0.75	(0.42, 1.33)	3.3 ± 4.1	0.98	(0.89, 1.09)
Congenital cataract	75	35 (46.7)	1.97	(1.17, 3.32)*	0.6 ± 0.7	1.45	(1.04, 2.02)*	4.0 ± 3.9	1.09	(1.02, 1.17)*
Cardiovascular
Common truncus	9	5 (55.6)	3.47	(0.78, 15.40)	0.6 ± 0.5	2.31	(0.68, 7.81)	3.2 ± 3.0	1.10	(0.85, 1.44)
Transposition of great arteries	68	21 (30.9)	0.81	(0.46, 1.45)	0.4 ± 0.6	0.86	(0.56, 1.30)	3.2 ± 3.7	0.99	(0.92, 1.07)
Tetralogy of Fallot	106	39 (36.8)	1.15	(0.73, 1.81)	0.4 ± 0.6	0.91	(0.67, 1.25)	3.2 ± 3.6	0.98	(0.92, 1.04)
Ventricular septal defect	1,579	489 (31.0)	0.98	(0.87, 1.11)	0.4 ± 0.7	0.97	(0.89, 1.05)	3.1 ± 3.7	0.99	(0.98, 1.01)
Atrial septal defect	822	258 (31.4)	0.91	(0.77, 1.08)	0.4 ± 0.7	0.93	(0.83, 1.03)	3.3 ± 4.0	0.99	(0.97, 1.01)
Endocardial cushion defect	43	14 (32.6)	1.18	(0.57, 2.43)	0.4 ± 0.7	1.13	(0.71, 1.80)	3.2 ± 3.9	1.01	(0.92, 1.11)
Pulmonary valve atresia/stenosis	457	145 (31.7)	1.04	(0.83, 1.30)	0.4 ± 0.7	1.07	(0.92, 1.24)	3.3 ± 4.0	1.01	(0.99, 1.04)
Tricuspid atresia/stenosis	44	17 (38.6)	1.78	(0.83, 3.81)	0.5 ± 0.8	1.40	(0.87, 2.24)	3.6 ± 4.8	1.05	(0.96, 1.14)
Ebstein’s anomaly	23	7 (30.4)	1.24	(0.41, 3.71)	0.4 ± 0.7	0.96	(0.46, 2.01)	2.7 ± 3.2	0.96	(0.82, 1.11)
Aortic valve stenosis	102	40 (39.2)	1.22	(0.78, 1.92)	0.5 ± 0.6	1.04	(0.76, 1.41)	3.4 ± 3.7	1.00	(0.94, 1.06)
Hypoplastic left heart syndrome	96	36 (37.5)	1.05	(0.65, 1.70)	0.5 ± 0.8	1.07	(0.79, 1.46)	3.8 ± 4.4	1.03	(0.97, 1.09)
Patent ductus arteriosus (≥ 2,500 g)	566	180 (31.8)	0.96	(0.78, 1.19)	0.4 ± 0.7	0.95	(0.83, 1.09)	3.2 ± 4.0	0.98	(0.96, 1.00)
Coarctation of aorta	235	65 (27.7)	0.94	(0.67, 1.30)	0.4 ± 0.7	1.04	(0.83, 1.29)	3.0 ± 3.8	1.01	(0.97, 1.05)
Craniofacial
Choanal atresia	99	28 (28.3)	0.77	(0.48, 1.26)	0.4 ± 0.7	0.93	(0.66, 1.31)	2.7 ± 3.7	0.96	(0.90, 1.03)
Cleft palate without cleft lip	340	110 (32.4)	1.14	(0.88, 1.48)	0.4 ± 0.6	0.99	(0.82, 1.19)	3.1 ± 3.6	1.01	(0.97, 1.04)
Cleft lip ± cleft palate	501	163 (32.5)	0.94	(0.76, 1.17)	0.4 ± 0.7	0.98	(0.85, 1.13)	3.4 ± 4.1	1.00	(0.97, 1.02)
Genitourinary
Renal agenesis/hypoplasia	174	61 (35.1)	1.13	(0.79, 1.62)	0.4 ± 0.6	1.02	(0.79, 1.30)	3.2 ± 3.6	1.00	(0.95, 1.04)
Musculoskeletal
Upper limb reduction	105	3,412	29 (27.6)	0.90	(0.56, 1.45)	0.4 ± 0.7	1.02	(0.73, 1.42)	3.0 ± 3.7	1.01	(0.95, 1.08)
Lower limb reduction	85	2,754	31 (36.5)	1.36	(0.82, 2.25)	0.5 ± 0.7	1.23	(0.87, 1.75)	3.5 ± 4.1	1.05	(0.98, 1.12)
Gastroschisis	108	3,527	23 (21.3)	0.48	(0.28, 0.81)*	0.3 ± 0.6	0.63	(0.43, 0.92)*	2.8 ± 3.9	0.95	(0.90, 1.01)
Omphalocele	81	2,559	23 (28.4)	0.93	(0.54, 1.61)	0.3 ± 0.5	0.83	(0.55, 1.25)	2.8 ± 3.3	0.98	(0.91, 1.05)
aHeat wave (≥ 3 consecutive days with daily mean UAT above the 90th percentile). bORs were adjusted for maternal age, race, ethnicity, adequacy of prenatal care, smoking, weather region, and year. *Statistically significant (p ≤ 0.05).																			

Estimates and 95% CIs from stratified analyses of heat waves by weather region, maternal race, ethnicity, age, education, prenatal care adequacy, and smoking status (data not shown) were imprecise and overlapping due to small sample sizes within strata. The only exception was associations between heat waves and cleft lip with or without cleft palate that varied significantly by ethnicity: Hispanic (aOR = 3.02; 95% CI: 1.44, 6.33) versus non-Hispanic (aOR = 0.83; 95% CI: 0.67, 1.05).

We performed a subanalysis among term infants to assess the potential influence of detection bias in preterm infants (data not shown). Adjusted ORs for congenital cataracts remained statistically significant: 5-degree increase in mean daily minimum UAT (aOR = 1.79; 95% CI: 1.28, 2.51), 5-degree increase in mean daily mean UAT (aOR 1.76; 95% CI: 1.25, 2.46), 5-degree increase in mean daily maximum UAT (aOR = 1.76; 95% CI: 1.25, 2.46), heat wave (aOR = 1.96; 95% CI: 1.09, 3.51), number of heat waves (aOR = 1.52; 95% CI: 1.05, 2.19), and number of days above the 90th percentile (aOR = 1.11; 95% CI: 1.03, 1.20). Associations with mild heart defects were similar to the full analyses, with no statistically significant associations.

**Figure 2:**
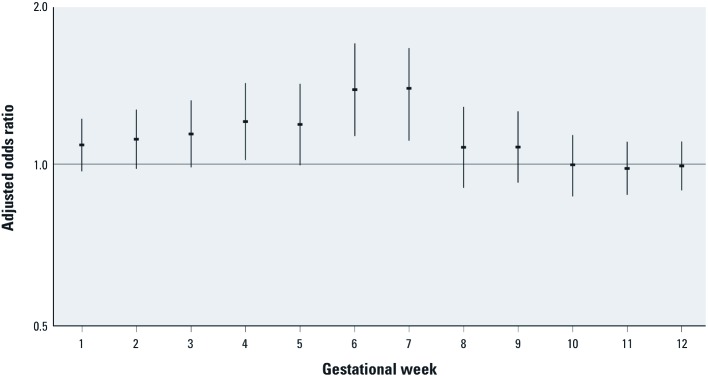
Associations between the mean daily minimum universal apparent temperature and congenital cataract (*n *= 75) by gestational week in the first trimester. Error bars represent 95% CIs.

We further evaluated associations with congenital cataracts according to gestational week in the first trimester (Figure 2). Adjusted ORs for mean daily minimum UAT and congenital cataracts were significantly positive for weeks 4, 6, and 7, and the overall pattern suggests increasing susceptibility from week 1 to weeks 6 and 7, and no association with exposure by week 10.

## Discussion

In our population-based study of 15 years of birth defects data in upstate New York, we found elevated ORs for associations between multiple indicators of increased summer temperature and extreme heat during the critical period of embryogenesis and congenital cataracts. We also observed inconsistent positive associations between some heat indicators and renal agenesis/dysplasia, and inconsistent negative associations for some heat indicators with anophthalmia and gastroschisis.

We did not see associations with other birth defects that have been associated with maternal hyperthermia in the animal and human literature. In our literature review of birth defects related to hot environments in early pregnancy, we found one study of neural-tube defects and two of congenital heart defects. Cooking in a hot kitchen; working, walking, or running in the sun; or lack of air conditioner use at home or work was not associated with neural-tube defects in a case–control study of 175 Mexican-American mothers of infants with neural-tube defects and 221 mothers with normal live births in a Texas–Mexico border population (Suarez et al. 2004). Congenital heart defects were not associated with exposure to temperatures > 100°F or hours per week of extreme temperatures in a previous population-based, case–control study of New York residents which interviewed mothers of 502 infants with heart defects and 1,066 infants without malformations (Judge et al. 2004). Workplace temperature > 20°C (68°F) was not associated with congenital heart defects in a case–control study of 573 mothers of infants with heart defects and 1,055 mothers with healthy infants in Finland (Tikkanen and Heinonen 1991). The exposure assessments for these studies were based on postpartum maternal interviews about environmental conditions during early pregnancy.

*Congenital cataracts.* Congenital cataracts are opacifications of the lens of the eye that interfere with normal development of vision in infants and are a leading cause of preventable blindness and visual impairment in children (Gilbert and Foster 2001). Known risk factors include genetic disorders, metabolic disorders, and maternal infections (e.g., rubella) (Gilbert and Foster 2001; Vogt et al. 2005). In the Hungarian Case–Control Study, anti-fever therapy attenuated the association between acute maternal infection with fever and congenital cataracts, suggesting that maternal fever may be the cause rather than the underlying infection, but no other studies have reported a direct association between maternal fever and cataracts (Vogt et al. 2005).

Our findings of consistent and statistically significant associations between the occurrence of congenital cataracts and heat exposures during the critical period, particularly for exposures during weeks 4–7, are consistent with both human and animal data. In humans, the developing eye appears on gestational day 22 (Sadler 2004). The lens placodes form the lenses of the eyes in week 5, and the primary lens fibers reach the anterior wall of the lens vesicle by the end of week 7. Weeks 4–7 are the most susceptible period for the development of congenital cataracts in women with rubella infection during pregnancy, whereas the lens is not affected by exposure after week 7 (Sadler 2004).

*Other birth defects.* Although microphthalmia and abdominal wall defects have been associated with hyperthermia in human and animal studies (Edwards 2006; Graham et al. 1998), anophthalmia/microphthalmia and gastroschisis showed statistically significant negative associations with certain heat indicators in our study. These negative associations, however, were not consistent across heat indicators and may have been due to chance.

Given the weight of evidence supporting the teratogenic effects of hyperthermia in animals and humans, and that the central nervous system has been demonstrated to be the most sensitive to hyperthermic insult (Edwards 2006; Edwards et al. 1995; Graham 2005; Graham et al. 1998; Warkany 1986), we expected to see elevated odds ratios for central nervous system defects. Nevertheless, we did not find evidence of associations in our population, and neither did Suarez et al. (2004) in their study of exposures to hot environments and the occurrence of neural-tube defects. However, because the CMR collects data on only major structural congenital malformations identified during the first 2 years of life, associations between temperature and outcomes reflecting less extreme central nervous system damage (e.g., neurobehavioral abnormalities) could not be examined in our study. In addition, the CMR does not collect data on elective terminations, spontaneous abortions, or stillbirths (NBDPN 2010), which can bias findings for major birth defects that may be detected and terminated or that may result in miscarriage or fetal death (Cragan et al. 1995; Parker et al. 2010). It is known that a large proportion of embryos with neural-tube defects perish *in utero* (Edwards et al. 1995).

*Potential teratogenic mechanisms of hyperthermia.* In human clinical studies, an oral temperature of 37°C (98.6°F) is considered normal, and 39°C (102°F) is regarded as the threshold for potential damage. Heat is teratogenic only when the temperature elevation coincides with a susceptible stage of development; and the type of defect depends on the particular stage of development (Edwards 1998, 2006; Edwards et al. 1995; Graham et al. 1998). Potential teratogenic mechanisms include cell death, disruption of the normal sequence of gene activity during organogenesis, and vascular disruption (Edwards 1998, 2006; Edwards et al. 1995; Graham et al. 1998).

*Study strengths.* Our study is one of the first to address the associations between extreme high environmental temperatures and the occurrence of birth defects. We analyzed 15 years of CMR and birth certificate data for a large geographically and demographically diverse population. In addition, we examined associations with multiple birth defect groups based on surveillance definitions proposed by the NBDPN (2010), which will allow future comparisons and collaborations with other states participating in the National Environmental Public Health Tracking Network (Centers for Disease Control and Prevention 2011b). Exposures during relevant developmental time windows were defined using objective meteorologic data, in contrast with previous studies that classified exposures based on maternal recall months to years after birth, potentially resulting in misclassification due to poor memory and recall bias (Judge et al. 2004; Suarez et al. 2004; Tikkanen and Heinonen 1991). We also used UAT, a better indicator of thermal stress on the human body than unadjusted temperature (Steadman 1984), and examined multiple heat indicators to capture potential effects of both intensity and duration. To account for potential acclimatization, we used a relative measure of extreme heat according to regional distributions of UAT.

*Study limitations.* Our exposure assessment was based on regional ambient temperatures measured at airport weather stations and maternal residence at birth. However, heterogeneity of outdoor and indoor temperature influenced by the built environment, time and activity patterns (e.g., time spent inside and outside at home, at work, in recreation), and thermoregulating behaviors (e.g., air conditioner use, hydration, removal from the hot environment) were not taken into account. Several studies have shown that air conditioner use modifies estimated effects of heat-related mortality (Anderson and Bell 2009; Medina-Ramón and Schwartz 2007; O’Neill et al. 2005). Due to inadequate geographic coverage and sample size, we were unable to incorporate air conditioner use data from the American Housing Survey (U.S. Census Bureau 2011) to perform a sensitivity analysis. We plan to add a question about air conditioner use to the New York Behavioral Risk Factor Surveillance System (New York State Department of Health 2011) to address this issue in future studies. Exposures in women who changed residence during pregnancy may have been misclassified. Because pregnant women may self-modulate their exposures to extremes in environmental heat when they become uncomfortable, maternal core body temperatures may not exceed the threshold necessary for teratogenesis. Chambers (2006) discussed this type of self-limiting behavior in pregnant sauna bathers. As described above, the CMR does not ascertain cases of birth defects among miscarriages, stillbirths, or elective terminations, which can bias findings for major birth defects that are more likely to be terminated or result in fetal death. We cannot rule out bias due to unmeasured confounding.

Last, because we performed multiple tests to examine the relationships between 28 birth defects groups and various heat exposure indicators in this hypothesis-generating study, statistically significant findings may have been attributable to chance. Under the null hypothesis, we would expect 4 of the 84 effect estimates displayed in Table 3 to be statistically significant at the *p* = 0.05 level. Thus, significant positive and negative associations with cataracts, renal agenesis, and anophthalmia may have been chance findings. Bonferroni adjustment to the *p* = 0.05 level of significance (0.05/84 = 0.0006) would yield approximate adjusted CIs for congenital cataracts that include the null value (95% CI: 0.93, 2.44). However, the associations with congenital cataracts are biologically plausible, particularly given stronger associations during the relevant developmental window of lens development, and associations were consistent across exposure metrics, making chance a less likely explanation for these findings.

*Conclusions.* We found positive and consistent associations with congenital cataracts of multiple ambient heat exposure indicators, including 5-degree increases in the mean daily UAT (minimum, mean, and maximum), a heat wave episode, the number of heat waves, and the number of days above the 90th percentile of UAT. Furthermore, these associations were strongest during the most relevant developmental window for congenital cataracts. Given our findings, associations with maternal fever reported by Vogt et al. (2005) and with hyperthermia in animals (Edwards 2006; Edwards et al. 1995; Graham 2005; Graham et al. 1998; Warkany 1986), the potential for maternal hyperthermia to cause congenital cataracts, a leading cause of avoidable blindness and visual impairment in children, should be more closely examined. We did not see consistent elevations in the ORs for other major structural birth defects, which is reassuring. However, our findings for congenital cataracts must be confirmed in other study populations.

## References

[r1] Anderson BG, Bell ML (2009). Weather-related mortality: how heat, cold, and heat waves affect mortality in the United States.. Epidemiology.

[r2] Centers for Disease Control and Prevention (2011a). International Classification of Diseases, 9th Revision, Clinical Modification.. http://www.cdc.gov/nchs/icd/icd9cm.htm.

[r3] Centers for Disease Control and Prevention (2011b). National Environmental Public Health Tracking Network Homepage.. http://ephtracking.cdc.gov/showHome.action.

[r4] Chambers CD (2006). Risks of hyperthermia associated with hot tub or spa use by pregnant women.. Birth Defects Res A Clin Mol Teratol.

[r5] Chinery R, Walker R (2009). Development of exposure characterization regions for priority ambient air pollutants.. Hum Ecol Risk Assess.

[r6] Cragan JD, Roberts HE, Edmonds LD, Khoury MJ, Kirby RS, Shaw GM (1995). Surveillance for anencephaly and spina bifida and the impact of prenatal diagnosis—United States, 1985–1994.. MMWR CDC Surveill Summ.

[r7] Czeizel AE, Puhó EH, Acs N, Bánhidy F (2007). High fever-related maternal diseases as possible causes of multiple congenital abnormalities: a population-based case-control study.. Birth Defects Res A Clin Mol Teratol.

[r8] Dlugosz L, Vena J, Byers T, Sever L, Bracken M, Marshall E (1992). Congenital defects and electric bed heating in New York State: a register-based case-control study.. Am J Epidemiol.

[r9] Edwards MJ (1998). Apoptosis, the heat shock response, hyperthermia, birth defects, disease and cancer. Where are the common links?. Cell Stress Chaperones.

[r10] Edwards MJ (2006). Review: Hyperthermia and fever during pregnancy.. Birth Defects Res A Clin Mol Teratol.

[r11] Edwards MJ, Shiota K, Smith MS, Walsh DA (1995). Hyperthermia and birth defects.. Reprod Toxicol.

[r12] Graham JM (2005). Marshall J. Edwards: discoverer of maternal hyperthermia as a human teratogen.. Birth Defects Res A Clin Mol Teratol.

[r13] Graham JM, Edwards MJ, Edwards MJ (1998). Teratogen update: gestational effects of maternal hyperthermia due to febrile illnesses and resultant patterns of defects in humans.. Teratology.

[r14] Gilbert C, Foster A (2001). Childhood blindness in the context of VISION 2020—the right to sight.. Bull WHO.

[r15] Hashmi SS, Gallaway MS, Waller DK, Langlois PH, Hecht JT, National Birth Defects Prevention Study (2010). Maternal fever during early pregnancy and the risk of oral clefts.. Birth Defects Res A Clin Mol Teratol.

[r16] Honein MA, Paulozzi LJ (1999). Birth defects surveillance: assessing the “gold standard.”. Am J Public Health.

[r17] Intergovernmental Panel on Climate Change (2007). Climate Change 2007: Synthesis Report. Contribution of Working Groups I, II and III to the Fourth Assessment Report of the Intergovernmental Panel on Climate Change (Core Writing Team, Pachauri RK, Reisinger, A, eds.).

[r18] Judge CM, Chasan-Taber L, Gensburg L, Nasca PC, Marshall EG (2004). Physical exposures during pregnancy and congenital cardiovascular malformations.. Paediatr Perinat Epidemiol.

[r19] Luber G, McGeehin M (2008). Climate change and extreme heat events.. Am J Prev Med.

[r20] Medina-Ramón M, Schwartz J (2007). Temperature, temperature extremes, and mortality: a study of acclimatization and effect modification in 50 United States cities.. Occup Environ Med.

[r21] Milunsky A, Ulcickas M, Rothman KJ, Willett W, Jick SS, Jick H (1992). Maternal heat exposure and neural tube defects.. JAMA.

[r22] Moretti ME, Bar-Oz B, Fried S, Koren G (2005). Maternal hyperthermia and the risk for neural tube defects in offspring: systematic review and meta-analysis.. Epidemiology.

[r23] National Center for Atmospheric Research (2009). https://ncar.ucar.edu/home.

[r24] National Climatic Data Center (2009). U.S. Climate Divisions webpage.. http://www.ncdc.noaa.gov/temp-and-precip/us-climate-divisions.php.

[r25] NBDPN (National Birth Defects Prevention Network) (2010). Selected birth defects data from population-based birth defects surveillance programs in the United States, 2003–2007.. Birth Defects Res A Clin Mol Teratol.

[r26] New York State Department of Health (2011). Behavioral Risk Factor Surveillance System Homepage.. http://www.health.ny.gov/statistics/brfss/.

[r27] O’Neill MS, Zanobetti A, Schwartz J (2005). Disparities by race in heat-related mortality in four US cities: the role of air conditioning prevalence.. J Urban Health.

[r28] Oster ME, Riehle-Colarusso T, Alverson CJ, Correa A (2011). Associations between maternal fever and influenza and congenital heart defects.. J Pediatr.

[r29] Parker SE, Mai CT, Canfield MA, Rickard R, Wang Y, Meyer RE (2010). Updated national birth prevalence estimates for selected birth defects in the United States, 2004–2006.. Birth Defects Res A Clin Mol Teratol.

[r30] Prakalapakorn SG, Rasmussen SA, Lambert SR, Honein MA, National Birth Defects Prevention Study (2010). Assessment of risk factors for infantile cataracts using a case-control study: National Birth Defects Prevention Study, 2000–2004.. Ophthalmology.

[r31] Roohan PJ, Josberger RE, Acar J, Dabir P, Feder HM, Gagliano PJ (2003). Validation of birth certificate data in New York State.. J Community Health.

[r32] Sadler TW (2004). Langman’s Medical Embryology. 9th ed.

[r33] Shaw GM, Nelson V, Todoroff K, Wasserman CR, Neutra RR (1999). Maternal periconceptional use of electric bed-heating devices and risk for neural tube defects and orofacial clefts.. Teratology.

[r34] Steadman RG (1984). A universal scale of apparent temperature.. J Climate Appl Meteor.

[r35] Suarez L, Felkner M, Hendricks K (2004). The effect of fever, febrile illnesses, and heat exposures on the risk of neural tube defects in a Texas-Mexico border population.. Birth Defects Res A Clin Mol Teratol.

[r36] Tanner K, Sabrine N, Wren C (2005). Cardiovascular malformations among preterm infants.. Pediatrics.

[r37] Tikkanen J, Heinonen OP (1991). Maternal hyperthermia during pregnancy and cardiovascular malformations in the offspring.. Eur J Epidemiol.

[r38] U.S. Census Bureau (2011). American Housing Survey Homepage.. http://www.census.gov/housing/ahs/.

[r39] Vogt G, Puhó E, Czeizel AE (2005). Population-based case-control study of isolated congenital cataract.. Birth Defects Res A Clin Mol Teratol.

[r40] Wang Y, Cross PK, Druschel CM (2010). Hospital discharge data: can it serve as the sole source of case ascertainment for population-based birth defects surveillance programs?. J Public Health Manag Pract.

[r41] Warkany J (1986). Teratogen update: hyperthermia.. Teratology.

